# Utilizing cocoyam (*Xanthosoma sagittifolium*) for food and nutrition security: A review

**DOI:** 10.1002/fsn3.602

**Published:** 2018-03-13

**Authors:** Abena A. Boakye, Faustina Dufie Wireko‐Manu, Ibok Oduro, William O. Ellis, María Gudjónsdóttir, Ioannis S. Chronakis

**Affiliations:** ^1^ Department of Food Science and Technology Kwame Nkrumah University of Science and Technology Kumasi Ghana; ^2^ DTU‐Food Nano‐Bio Science Research Group Technical University of Denmark Lyngby Denmark; ^3^ Faculty of Food Science and Nutrition University of Iceland Reykjavík Iceland

**Keywords:** adaptable technologies, cocoyam, food use, *Xanthosoma sagittifolium*

## Abstract

The critical role of indigenous crops in the socioeconomic growth of developing nations has necessitated calls for accelerated exploitation of staples. Cocoyam, *Xanthosoma sagittifolium,* is food for over 400 million people worldwide and is the most consumed aroid in West Africa. However, it remains an underexploited food resource. This study reviews existing literature and also makes use of primary data from interviews with indigenous cocoyam farmers, processors, consumers, and cocoyam scientists in the research Institutes of Ghana, to provide insight into existing nomenclature of the species, indigenous knowledge on food uses, nutritional value, and potential novel food applications of cocoyam. Adaptable technologies in conformity to new trends in food science that could be employed for in‐depth molecular studies and further exploitation of the crop are also discussed. It is envisaged that the provided information would contribute to global efforts aimed at exploiting the full potential of indigenous crops for sustainable food and nutrition security.

## 1 INTRODUCTION

Cocoyam (*Xanthosoma sagittifolium)* is among the world's six most important root and tuber crops (FAO, 2012). It is pantropical and has been domesticated in most communities in Oceania, Africa, and Asia (Ramanatha, Matthews, Eyzaguirre, & Hunter, 2010) providing sustenance for over 400 million people (Onokpise et al., 1999; Vaneker & Slaats, 2013). Africa is the major producer with West and Central Africa, notably, Nigeria, Ghana, and Cameroon contributing to over 60% of the total African production (Onyeka, 2014). Thus, the importance of cocoyam to regional food security cannot be overstated.

In spite of its high productivity levels and better storability compared to other tropical root and tuber crops (Opara, 2003; Opoku‐agyeman, Bennet‐Lartey, & Markwei, 2004; Quaye, Adofo, & Nimoh, 2010; Ramanatha et al., 2010), *Xanthosoma sagittifolium* has been marginalized in agricultural policies and research interventions on root and tuber crops. It remains an underexploited food resource (Falade & Okafor, 2013; Onyeka, 2014), with a reported decline of production levels in Ghana and Cameroon (Onokpise et al., 1999; Onyeka, 2014).

The challenge of underutilization is exacerbated by the existing confusion in taxonomy and nomenclature which limits researchers in exploiting data from one area of indigene to another (CABI 2014; Crop Trust 2010; Osuji & Nwala, 2015; Vaneker & Slaats, 2013). This makes it essential to provide an overview of the taxonomic reference and employed nomenclature in every scientific study for accurate information dissemination and use.

**Figure 1:**
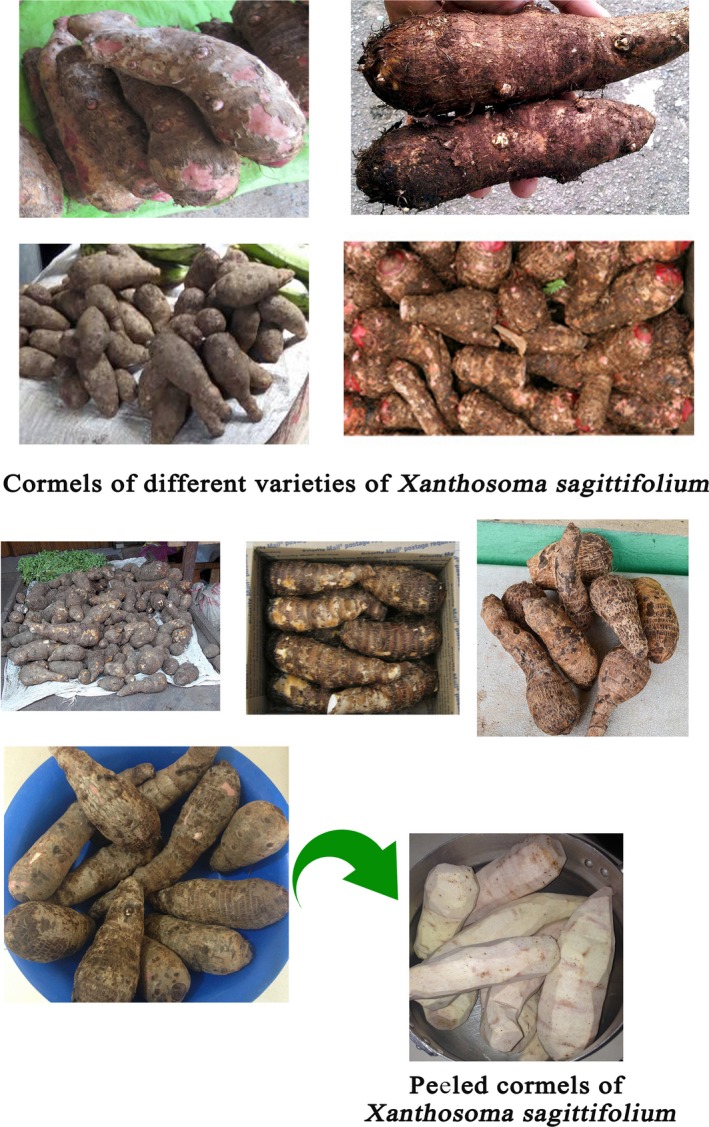
Different varieties of *Xanthosoma sagittifolium* cormels. Credit: Authors’ pictures and Google images

A comprehensive insight into existing indigenous varieties and the potential utilization of these varieties, as well as adaptable modern analytical methods for research in sub‐Saharan Africa, would facilitate efforts to enhance the utilization of these indigenous varieties for sustained food and nutrition security (Figure 1).

The aim of this review was therefore to look at existing work or literature available, giving an overview of the existing taxonomy and knowledge for *Xanthosoma sagittifolium,* provide insight into its production trends in Ghana, discuss the existing food uses in the West African subregion, and propose potential technologies that could be employed for in‐depth studies on their food properties to enhance the food utilization of the crop. Primary data from interviews with indigenous cocoyam farmers and cocoyam scientists in the Research Institutes of Ghana, namely, the Crops Research Institute and the Food Research Institute also contributed greatly to this review.

## 2 ORIGIN AND HISTORY OF COCOYAM IN GHANA

*Xanthosoma* spp. originate from tropical America (Crop Trust 2010); most probably, Central and South America (Ramanatha et al., 2010), where the species are believed to have been domesticated from the wild (Bermejo & León, 1994). *Xanthosoma* spp. are listed as invasive in many areas of the world (French Polynesia, Florida, the Galápagos Islands, Puerto Rico, and Costa Rica) in addition to being intentionally introduced to several other regions including Africa and Asia (Crop Trust 2010). It is a more recent introduction to Ghana and West Africa compared with the other prominent genus, *Colocassia*, which is speculated as being native to the subregion (Doku, 1966), since there is no known period of introduction of the crop. *Xanthosoma* is therefore referred to as “new cocoyam” in West Africa. Brown (2000) traced its introduction to West Africa as far as the 16th and 17th centuries, but Wright (1930) reported its introduction to Ghana in 1843 (19th century) by the West Indian Missionaries: There is paucity of information on the species in the region between the 17th and 19th centuries.

*Xanthosoma* is believed to have been first planted in Akropong Akwapim in the Eastern Region of Ghana. It is convenient as a cover crop, due to the plants large leaves, for seedlings of cocoa, an important cash crop and this led to its gradual spread through the forest belt of the country notably, the Ashanti, Western and Brong‐Ahafo regions as it was cultivated on cocoa plantations (Doku, 1966; Ramanatha et al., 2010). To date, *Xanthosoma* is an important crop in the humid areas of southern Ghana, ranking third to cassava and yam as important national root and tuber staple.

## 3 TAXONOMY AND NOMENCLATURE OF COCOYAM

Cocoyam, in most literature, is a collective name for species of the two most cultivated genera, *Colocasia* and *Xanthosoma,* of the edible aroids from the family Araceae (Opara, 2003; Ramanatha et al., 2010). Both genera have diverse species and a wide geographical distribution, spanning the tropical and subtropical regions of Oceania, Asia, and Africa. Thus, each have several local, traditional, and scientific names (CABI 2013). This, coupled with the morphological similarities between species in a genera, has contributed to the confusion in the use of terminologies for their identification (CABI 2013; Vaneker & Slaats, 2013).

*Colocasia* has 11 – 16 identified species (CABI 2013; Long & Liu, 2001) with the most common, *Colocasia esculenta*, being ascribed with two botanical varieties, *Colocasia esculenta* var *esculenta* (commonly referred to as dasheen) and *Colocasia esculenta* var *antiquorium* (commonly referred to as eddoe). The two are commonly referred to as taro and old cocoyam in most communities of West Africa (Doku, 1966). However, this nomenclature has been challenged in recent years with some botanists referring to the two varieties as different species (CABI 2013; Crop Trust 2010; Opara, 2003; Ramanatha et al., 2010). Thus, there is the need for a taxonomic review of the species to facilitate the dissemination and use of scientific data on the genera.

The genus, *Xanthosoma*, has been ascribed with 50–60 species (Stevens, 2012), and all cultivated varieties are currently grouped under four species: *X*. *sagittifolium, X*. *caracu, X*. *atrovirens,* and *X. nigrum (X. violaceum)* (CABI 2014; FAO 2013). Of these, the two most cultivated and economically important ones are *X*. *sagittifolium* and *X. nigrum* (Vaneker & Slaats, 2013). The foregoing classification is however disputable as some identified species cannot be put under any of the four groups (FAO 2013). This further necessitates the need for a taxonomic review. For simplicity, it is the norm for researchers to refer to all clones of cultivated edible *Xanthosoma* spp. as *X*. *sagittifolium* (FAO 2013), posing a hindrance to accurate dissemination and use of scientific data on the genus.

The original range of the genus is uncertain (CABI 2014). It is however generally agreed to be highly versatile in its requirements for growth and ease of adaptation to new locations making it an optimal choice crop for many climates (CABI 2014; Vaneker & Slaats, 2013), and a potential food security measure for developing economies.

**Figure 2:**
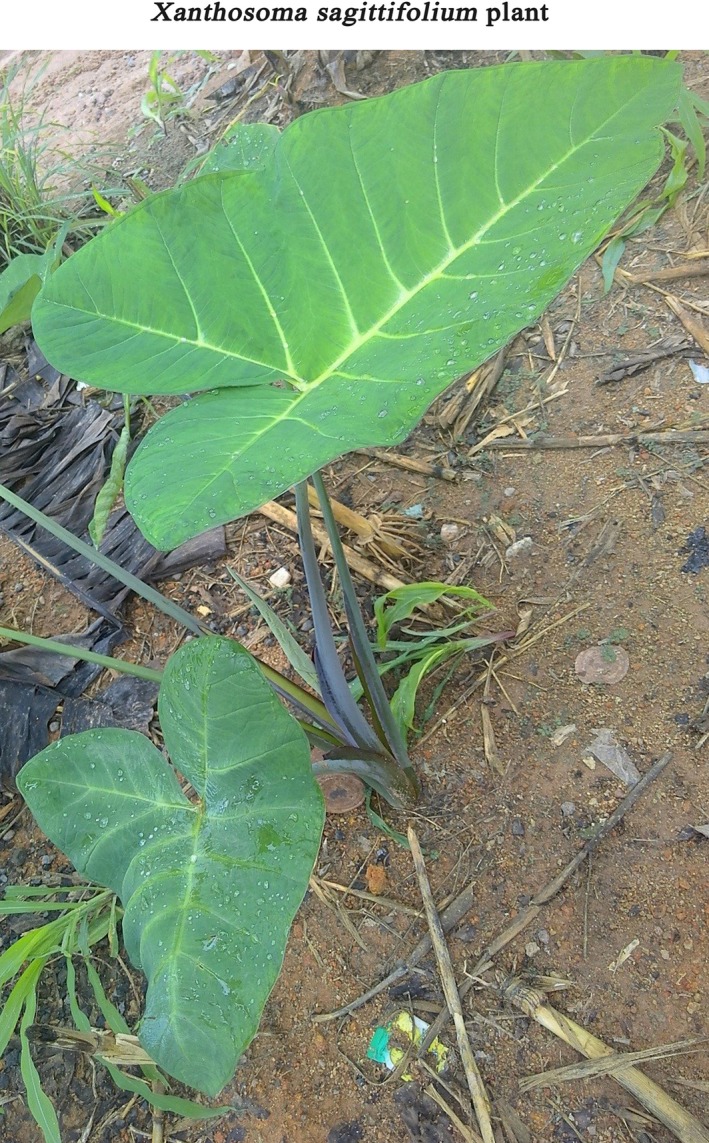
A young *Xanthosoma sagittifolium* (L.) Schott plant. Credit: Authors’ picture

The existing confusion on its taxonomy and nomenclature, however, is a major drawback to utilization of available scientific data from different areas of indigene in seeking to tap the full potential of *Xanthosoma* spp. (Crop Trust 2010; Ramanatha et al., 2010). To date, any meaningful study on its food use must be assessed on the basis of available species in a given location without researchers having the liberty to accurately exploit existing data from other studies as is commonly performed for other root and tuber crops. In spite of *Xanthosoma* spp. being the main edible aroid in West Africa (Opara, 2003), and Ghana in particular (Acheampong, Osei‐adu, Amengo, & Sagoe, 2015; Ramanatha et al., 2010), there is a dearth of studies on the properties of indigenous cultivated varieties to inform its industrial application and food use (Acheampong et al., 2015; Opara, 2003; Ramanatha et al., 2010) (Figure 2).

Notwithstanding the confusion in the taxonomy and nomenclature, *Colocasia esculenta* (L.) Schott is largely referred to as taro and *Xanthosoma sagittifolium* (L.) Schott as tan(n)ia, and the two are called cocoyam (s) (CABI 2013; Crop Trust 2010; Doku, 1966; Opara, 2003; Ramanatha et al., 2010). In West Africa, *Colocasia* spp. is called “old cocoyam/yam” and *Xanthosoma* spp. is called “new cocoyam/yam” because the former is said to be native to the region whereas the latter was introduced (Doku, 1966; Karikari, 1979). For the purposes of this review, the use of the word cocoyam refers to *Xanthosoma sagittifolium* (L.) Schott.

## 4 COCOYAM PRODUCTION TRENDS: GHANA IN FOCUS

Cocoyam has largely been cultivated on a subsistence basis since its introduction to Ghana. It served as a cover crop for seedlings of cocoa, and eventually spread through the forest belt along with cocoa plantations (Doku, 1966; Karikari, 1979). Due to its ease of adaptation to diverse habitats (Manner, 2011), exceptional keeping qualities of the cormels (Doku, 1966) and prolific sprouting after clearing and burning of secondary forests, cocoyam has become an important agricultural commodity. It was one time ranked the most important staple root crop in Ghana with estimated production level of 509.6 thousand tons as opposed to 504.2 thousand tons of cassava, the closest competitor (Karikari, 1979).

However, there were no concerted efforts to promote the growth and performance of cocoyam relative to other root and tuber crops until the outbreak of the root rot complex disease in 1925 after which two formal studies on the species were conducted in Ghana (Doku, 1966; Wright, 1930). There was a lack of information and follow‐up studies on domesticated varieties until 1971 when Karikari carried out a germplasm assessment and collection (Karikari, 1971). This was then followed with an evaluation of the susceptibility of local and exotic varieties to the root rot complex disease (Karikari, 1979). Similar individual studies were also carried out (Offei, Asante, & Danquah, 2004; Opoku‐agyeman et al., 2004; Safo‐Kantanka & Adofo, 2007; Tortoe & Clerk, 2011) but with an absence of a common front to purposefully enhance the growth and performance of cocoyam (Quaye et al., 2010; Ramanatha et al., 2010).

**Figure 3:**
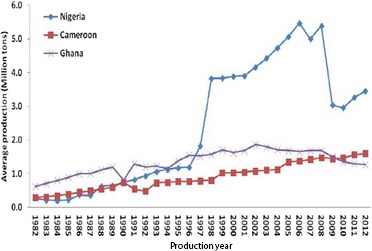
Cocoyam production trend in Ghana compared with the two other major producers in sub‐Saharan Africa. Credit: Onyeka (2014)

The marginalization in agricultural policies and research interventions left the cultivation of the crop largely in the hands of resource‐poor, rural farmers who undertook selective cultivation of varieties to the detriment of the already limited germplasm (Ramanatha et al., 2010). At present, there are only two commercially cultivated farmer varieties (Acheampong et al., 2015), cocoyam now ranks third in importance after cassava and yam (Acheampong et al., 2015; Ramanatha et al., 2010), and there has been a steady decline in its production levels since 2009 (Figure 3).

The reduced production has reflected in a drop in cocoyam exports from 242 thousand tons in 2009 to 33 thousand tons in 2012 (Appiah, 2016). The Council for Scientific and Industrial Research (CSIR) of Ghana in 2012, released three new varieties, namely *Mayɜyie* (white variety), *Akyɜdeɜ* (red variety), and *Gyemedi* (red variety) with improved pest and disease resistance, shorter maturity, and enhanced cooking quality. These improved varieties are yet to be cultivated by farmers (Acheampong et al., 2015). Other confounding constraints to optimal production of cocoyam include decreasing rainfall and soil fertility, loss of forests, weak technical, and institutional support as well as high cost of inputs (Acheampong et al., 2015; Quaye et al., 2010).

With a prevailing production of 5–7.5 tons per hectare against an estimated potential production of 23.5–35 tons per hectare (Onyeka, 2014), recent efforts to strengthen the performance of staple root and tuber crops have addressed some challenges of *Xanthosoma*. Notable among these interventions are the Root and Tuber Improvement and Marketing Programme (2007–2015), the West African Productivity Programme (2008–2018), and the DANIDA Strengthening Root and Tuber Value Chains Project (2013–2017). Cocoyam researchers still lament the many existing knowledge gaps that influence the performance of cocoyam utilization, and advocate for more interventions specifically tailored to promote the production, processing, and utilization of the crop (Acheampong et al., 2015).

## 5 EXISTING KNOWLEDGE AND USES OF COCOYAM

### 5.1 Existing knowledge on cocoyam

Cocoyam farmers remain the main custodians of production and varietal information of the crop in West Africa (Acheampong et al., 2015; Ramanatha et al., 2010). Most varieties currently cultivated in the subregion are believed to be landraces introduced by the Portuguese and or West Indian missionaries (Doku, 1966; Onokpise et al., 1999; Ramanatha et al., 2010). Currently, there are two commercially cultivated varieties (Acheampong et al., 2015) from the earlier reported 4–5 landraces in Ghana (Doku, 1966; Karikari, 1979), and three released but not yet commercially cultivated varieties (Acheampong et al., 2015). The landraces are generally distinguished by the color of the peeled cormels resulting in red, white, and yellow varieties with corresponding words in local dialects used for their identification (Doku, 1966; Onokpise et al., 1999). Locals also differentiate their cooking properties based on their cooked texture and required cooking time to give soft and firm/hard texture, as well as short‐ and long‐cooking varieties, respectively (Doku, 1966; Karikari, 1979). Available information on Nigerian landraces has advanced a step further in ascertaining the true taxa (Osuji & Nwala, 2015) and some processing properties of indigenous varieties (Falade & Okafor, 2013, 2014; Ojinnaka, Akobundu, & Iwe, 2009) but same is not true for Ghana and other West African countries. The knowledge gap on in‐depth processing properties of cultivated varieties is a major drawback to establishing the full potential of *Xanthosoma sagittifolium* through development of novel food products and other industrial uses.

### 5.2 Indigenous uses of cocoyam

Generally, all plant parts (cormels, petioles, leaves, and inflorescence) of cocoyam are edible (CABI 2014; Vaneker & Slaats, 2013). The wide distribution of the crop in different geographical areas and cultures has resulted in varying usage of the crop from one location to another (Vaneker & Slaats, 2013). They are mainly used as food in cultivated areas, and the plant parts are also used as fodder/feed and medicine, including its use as antipoisonous agents against tarantula, scorpion, and snake bites.

In its application as food, none of the plant parts are consumed raw because of its acridity (Doku, 1966; Ramanatha et al., 2010). Thus, most traditional cooking methods employ heat by boiling, baking, roasting, or frying, either alone or in combination with other ingredients to obtain delicacies (Lim, 2016; Opara, 2003). These may be snacks, main meals, or special dishes for vulnerable groups. In West Africa, there are significant similarities in the use of indigenous root and tuber crops (Falade & Okafor, 2014). However, cocoyam seems to be limited in this wise as only a handful of traditional dishes are popular across the major cocoyam areas in the region (Ghana, Nigeria, Cameroon, and Côte d'Ivoire), and Ghana seems to dominate in diversity of indigenous cocoyam dishes (Table 1).

**Table 1:** Indigenous food uses of cocoyam in some West African communities

Plant part	Name of dish/food	Method of preparation	Origin and/cultural sentiment	Improved product for convenience
Cormels	*Ampesi*	Peeled, boiled in water (can also be steamed). Served with sauce/stew	Ghana, Cameroon^a^^,^ b	None^e^
	*Akaw*	Smaller cormels, boiled with skin, and served with sauce. Skin removed before eating	Ghana^e^	None^e^
	*Fufu*	Peeled, boiled until cooked, and pounded into dough. Served with soup or stew depending on the geographical area	Ghana, Nigeria, Côte D'Ivoire^a^^,^ d ^,^ f	Packaged *fufu* Flour (cottage and industrial production)^a^
	*Ɛtͻ*	Peeled, boiled, and mashed with other ingredients into a one‐pot meal	Ghana^e^ Delicacy for all age groups but usually given to infants and the aged	None^e^
	*Ɔgͻͻ*	Roasted before peeling and mashed with other ingredients into a one‐pot meal	Ghana^e^ It is a lovers meal among some Akan clans in Ghana	None^e^
	Fried chips/crisps	Peeled, sliced, and fried in vegetable oil	Ghana, Cameroon, Nigeria, Cote D'Ivoire a ^,^ e	Few productions from a handful of Cottage industries especially during bumper season of the crop
	Soup thickener	Boiled, pounded, and put in soup while still on fire	Nigeria and Ghana^d^	Flour (Cottage production)^c^
	*Mpotompoto*	Peeled, cut into small chunks, boiled, and added to soup base to give a one‐pot meal	Ghana Delicacy for all age groups but usually given to infants and the aged	None^e^
	Ikokore	Grated, mixed with condiments, then steamed in leaves for 30 minutes	Nigeria	None^e^
Leaves	Palava sauce	Sliced, steamed/boiled, and added to sauce base	Ghana	None^e^
	Kontomire *abomuu*	Boiled, mashed with other ingredients (usually, pepper, onion, and fermented fish), and served with *ampesi*	Ghana	None^e^
	*Abunuabunu*	Boiled, mashed, and added to soup as thickener	Ghana	None^e^

Information was culled from ^a^Acheampong et al., 2015; ^b^Onokpise et al., 1999; ^c^CABI 2014; Doku, 1966; ^d^Falade & Okafor, 2014; ^f^Ramanatha et al., 2010 and ^e^authors’ personal communication from consumers in cocoyam‐growing areas of Ghana and review of available literature.

Indigenous dishes usually carry deep sociocultural sentiments and values (Ramanatha et al., 2010; Vaneker & Slaats, 2013). However, with the exception of few traditional delicacies that have been improved to meet growing consumer needs for convenience (Table 1), most dishes are prepared using traditional recipes and methods which are tedious and time‐consuming (Acheampong et al., 2015; Ramanatha et al., 2010). This is a major limitation to sustained consumption of these delicacies in an era of consumer demand for shelf‐stable, ready‐to‐prepare, and ready‐to‐eat (instant) dishes due to changing work schedules and responsibilities of women, who primarily are responsible for home cooking in most cultures of cocoyam cultivation.

The lack of convenient dishes/products from cocoyam also limits the exploitation of the crop in the international retail industry via supermarket chains. Preliminary studies by the authors have indicated a high consumer need for improvement and enhancement in the preparation processes and storability of these traditional dishes, especially in the urban settlements. The pressing need for research to improve these culturally acceptable dishes for convenience in preparation and trade cannot be overemphasized. Lessons can be learnt from the Asian food industry, where *Colocassia* spp. have been widely exploited. For example, high quality flours have been developed and commercialized for use in home‐made dishes and in various sweet desserts across the continent. Japan, in particular, has successfully expanded the use of cocoyam through the dissemination of novel and old recipes in popular media. As a result, the crop is held in high esteem even among the younger generations, ensuring its sustained utilization and trade, while simultaneously establishing its contribution to food and nutrition security in the region (Ramanatha et al., 2010).

## 6 NUTRITIONAL PROFILE OF COCOYAM

Malnutrition is a major challenge to the growth and productivity of tropical developing economies where most staples are carbohydrate‐rich but deficient in micronutrients. Cocoyam is postulated to have superior nutritional value over other major root and tuber staples of West Africa, especially in terms of their protein digestibility and mineral composition (Calcium, Phosphorous and Magnesium) (Chukwu, Ekwe, & Anyaeche, 2008; Ekwe, Nwosu, Ekwe, & Nwachukwu, 2009; Lim, 2016; Opara, 2003) (Table 2).

**Table 2:** Nutritional profile of cocoyam varieties from different geographical areas

Parameter	Mean values of 37 samples from Tonga and Papua New Guinea Cormels)^a^	Mean values from East Africa (Tanzania and Uganda; pooled data)^e^	Values of indigenous varieties from Nigeria (Cormels)^c^	Mean values of different portions (apical, middle and distal) of cormels from red and white varieties of cocoyam in Ghana^b^		Mean values form Cameroonian accessions^d^		
	Proximate (%)	Proximate (g/100 g)	Proximate (%)	Proximate (g/100 g)		Cormels	Leaves	Shoots
				Red	White			
Moisture	67.1	68.41	80.99	67.83	63.33			
Energy kJ 100 per g	521					133	34	33
Protein	1.55	4.75	5.47	4	5.12	2.0	2.5	3.1
Carbohydrate		20.95				31	5	5
Starch	27.6		11.03	22.83	27.2			
Sugar	0.42							
Crude fiber	0.99	1.96	1.28	1.54	1.39	1	2.1	3.2
Fat	0.11	0.43	0.2	0.59	0.43	0.3	1.6	0.6
Ash	1.04	3.51	1.03	3.2	2.55			
Minerals (mg/100 g)								
Calcium	8.5	110.17		13.51	12.15	20	95	49
Phosphorous	53	207.50		53.23	45.7	47	388	80
Magnesium	27	90.62		73.1	63.57			
Sodium	6.6	23.98		22.13	36.1			
Potassium	530	908.25		1248.33	1112			
Sulfur	7.9							
Iron	0.4	4.54		3.05	3.53	1	2	0.3
Copper	0.19	0.63						
Zinc	0.52	2.72		32.6	41.9			
Manganese	0.17	1.95						
Aluminum	0.53							
Boron	0.09							
Vitamins (mg/100 g)								
Vitamin A (ret. + ‐car./6)	0.005					Trace	3.3	
Thiamin	0.024					0.1		
Riboflavin	0.032					0.03		
Nicotinic acid	0.8					0.5		
Ascorbic acid						10	37	82
Pot. Nic. Acid = Trp/60	0.33							
Total vitamin C (AA + DAA)	14							

Source: ^a^Bradbury and Holloway (1988); ^b^Sefa‐Dedeh and Agyir‐Sackey (2004); ^c^Odebunmi, Oluwaniyi, and Bashiru (2010); ^d^Opara (2003), ^e^Ndabikunze et al. (2011). Blank space indicates data not provided by author(s).

According to Opara (2003), *Xanthosoma sagittifolium* can generally be regarded as an appreciable (middle range) source of dietary energy, proteins, and vitamins. It is said to be high in potassium, zinc, and nicotinic acid as well as a low inhibitor of trypsin compared to other edible aroids (Bradbury & Holloway, 1988).

This nutritional potential of cocoyam could be adequately tapped by diversifying its utilization through the development of new food products for different food industries and market needs. Two such industries which could be targeted are the snack and complementary (baby) foods sector. Both are currently thriving markets in sub‐Saharan Africa and utilizing cocoyam thus would go a long way to produce culturally acceptable and nutritious food products to enhance the variation of products for sustained food and nutrition security. Cocoyam leaves are known to have appreciable levels of antioxidants, vitamins, and dietary fiber (Ekwe et al., 2009; Lebot, 2009), but these potential health benefits have barely been explored in the food and pharmaceutical industries.

### 6.1 Acridity and antinutritional properties of cocoyam

A major drawback to the food use of cocoyam is the presence of antinutrients, predominantly, oxalates in all of the plant parts (cormels, petioles and leaves) (Ramanatha et al., 2010; Sefa‐Dedeh & Agyir‐Sackey, 2004). Their presence interferes with the bioavailability of other nutrients and may be detrimental to human health when consumed beyond certain thresholds (Doku, 1966; Sefa‐Dedeh & Agyir‐Sackey, 2004). Also, if not well cooked, consumption of most parts especially cormels and leaves, cause irritable sensations (itching) in the throat (Sefa‐Dedeh & Agyir‐Sackey, 2002; Sefa‐Dedeh & Agyir‐Sackey, 2004). Thus, the need for some form of processing and/or treatment before consumption and other food use.

This attribute of cocoyam, however, should not necessarily hamper efforts at enhancing its utilization. This is because most staple crops require one treatment form or the other to render them safe for consumption. Common processing techniques/treatment methods employed for other staples in the subregion such as grating, soaking, steaming, and boiling have been demonstrated to be effective in reducing oxalate levels in cocoyam (Aniekwe, 2015; Doku, 1966; Ramanatha et al., 2010). It is noteworthy that the levels of oxalate vary with different cocoyam varieties (Doku, 1966; Ramanatha et al., 2010). As a result, each cultivated variety must be investigated to ascertain the oxalate composition for informed selection of processing techniques/treatment methods. Drying (oven, drum, sun, and solar) has also been established to be efficient in reducing the antinutrient content of cocoyam to appreciable levels (Amandikwa, 2012; Sefa‐Dedeh & Agyir‐Sackey, 2004). Fermentation has been shown to significantly reduce the levels of oxalates, phytates, and tannins in *Colocasia* spp. (Adane, Shimelis, Negussie, Tilahun, & Haki, 2013), and these processes could be replicated in indigenous *Xanthosoma* varieties for enhanced utilization.

## 7 POTENTIAL FOOD USES OF COCOYAM

Innovations in food product development have not taken advantage of all portions of cocoyam being edible and researchers to date, lament the limited utilization of cocoyam as a food resource, particularly, its use as a sustainable food security measure in developing economies (Acheampong et al., 2015; Crop Trust 2010; Falade & Okafor, 2013, 2014; Karikari, 1979; Lebot, 2009; Opara, 2003; Ramanatha et al., 2010). These are indications of the need to further exploit the nutritional advantages of the crop; primarily, its cormels, and then, leaves, petioles, and inflorescences through convenient foods.

### 7.1 Cormels (roots)

The cormels of cocoyam are of major economic importance compared with the other parts of the crop. However, its bulkiness and short shelf‐life is a major challenge to utilization. With the appropriate research on their processing properties, suitable technologies could be employed to resize and process cormels into shelf‐stable convenient (ready‐to‐prepare) chips and crisps as well as formulate new recipes for national and regional markets. The resized cormels could also be packaged as shelf‐stable ready‐to‐prepare *ampesi* to meet changing consumer needs while simultaneously providing new markets and improved livelihoods for cocoyam producers. The cormels also have the potential to be used in the beverage industry as adjuncts (Eneh, 2013).

According to Lebot (2009) and Ramanatha et al. (2010), industrial processing of the cormels into flours may be a viable economic outlet for storage and further use. The flours could be used in the snack and food industries by employing technologies such as extrusion‐cooking and drum drying to develop ready‐to‐prepare/ready‐to‐eat healthy snacks and complementary foods. They could also serve the general‐purpose market as improved soup/sauce thickeners (Falade & Okafor, 2014). Surprisingly, most indigenous delicacies of cocoyam (Table 1) have not been improved to ease preparation constraints, and so their patronage especially in urban areas is gradually declining. The flours may therefore be a good start for food scientists and technologists to improve on the dishes for convenience and extend shelf‐life. For instance ε*tͻ* and *mpotompoto,* local delicacies now seldom prepared in urban homes because of the tedious and time‐consuming preparation methods could be improved to minimize the preparation time. A preliminary study by the authors to improve the preparation process and storability of these two delicacies resulted in novel products that had very high acceptance and willingness to purchase in initial in‐house consumer assessments (Boakye et al., 2016).

Although some authorities have suggested good properties of starches from West African cocoyam varieties (Falade & Okafor, 2013; Sefa‐Dedeh & Agyir‐Sackey, 2002), they may not be economically viable to process for industrial use. This is because cocoyam cannot compete with the starch yields of cassava (a more abundant staple with higher starch yields) for industrial application. Thus, it may be more cost‐effective to consider the utilization of cocoyam starch only when it is a by‐product of another industrial process.

### 7.2 Leaves, petioles, and inflorescence

Some areas of cocoyam domestication use the petioles and inflorescence as condiments for soups and sauces (CABI 2014), but there is a lack of literature on their food use in the West African cuisine. Thus, in seeking to enhance culturally acceptable food uses of cocoyam, it would be expedient to focus on the already consumed and economically viable leaves. The cocoyam leaf is a delicacy in sauces and soups, especially for Ghanaian communities but remains underexploited. A major challenge to its utilization is the limited shelf‐life (3–5 days maximum) (Acheampong et al., 2015). However, the leaves could be processed into purees or flours for end uses such as in preparation of soups and sauces, as well as in novel products to tap their nutritional value. For example, to address the time constraints coupled with irritations on the skin associated with the preparation of *palaver sauce* (Table 1), studies on appropriate packaging techniques could be undertaken to package ready‐to‐use (pre‐sliced) leaves. Such provision(s) could enhance its use in urban cities and contribute to efforts aimed at meeting the growing consumer need for diversity and convenience. The product (s) may also be exported to serve the needs of expatriates from West Africa (and other cocoyam growing areas) who have to substitute their cocoyam leaves with other vegetables in preparation of *traditional delicacies* abroad. Moreover, the high nutritional value of cocoyam leaves (Table 2) could be explored in new markets in Europe and the United States of America, to substitute for other vegetables such as spinach, with such convenient product(s).

## 8 ADAPTABLE TECHNOLOGIES FOR CHARACTERIZATION OF COCOYAM

Traditionally, the use of conventional methods that assesses the macro‐ and microproperties of food matrices have been employed to propose food uses of the flours and starches of *Xanthosoma sagittifolium* (Falade & Okafor, 2013, 2014; Rašper, 1969a,b; Sefa‐Dedeh & Agyir‐Sackey, 2002, 2004). These methods are, however, limited in their assessment of molecular and structural characteristics of food materials thereby restricting their prediction of the final eating quality of the processed foods (Mortensen, Thybo, Bertram, Andersen, & Engelsen, 2005). There is also a paucity of information on the cooking properties of the cormels limiting their expanded use in the food industry.

New trends in food science advocate the use of sensitive but rapid and nondestructive technologies that require less sample preparation and can measure several attributes concurrently (Frosch, Dissing, Adler‐Nissen, & Nielsen, 2011; Huang, Liu, & Ngadi, 2014). Spectroscopy is one such avenue of interest in research. Advanced spectroscopy in food research, proposes the use of spectral imaging technology comprising both multi‐ and hyperspectral imaging for a more precise assessment of quality characteristics of heterogeneous matrices such as food (Dissing, Nielsen, Ersbøll, & Frosch, 2011; Frosch et al., 2011).

The technique employs both vision (imaging/camera) and spectral (spectroscopic) technologies to give combined spatial, spectral, and surface chemistry information of a sample (Feng & Sun, 2012; Frosch et al., 2011). Here, light reflection at a large number of different wavelengths is used to produce a spectral image from which both spectral and spatial information can be obtained simultaneously. Thus, minute details in texture, color, microstructure, and surface chemistry of a heterogeneous matrix could be obtained in one test (Andresen, Dissing, & Løje, 2013). This simultaneous measure of different attributes in a single test positions the technology as an ideal cost‐effective technology for the ever‐changing and demanding food industry (Feng & Sun, 2012). Details on the modus operandi, application of the technology in the food industry, and potential utilization have been reported by other researchers. The two procedures, multispectral and hyperspectral imaging, differ only in the number of bands involved, over 100 for *hyper* and about 10 for *multi* spectral imaging. This gives multispectral imaging a competitive advantage over hyperspectral imaging in ease of data processing and practicality in industrial online applications (Feng & Sun, 2012).

Another spectroscopic technique now popular in food analysis is Low Field ^1^H (and High Field) Nuclear Magnetic Resonance (NMR) analysis (Mortensen et al., 2005). The tool has the unique advantage of measuring the bulk of the sample while its spectroscopic data correlate to individual nuclei in a molecule (Hansen et al., 2010). The technique being nondestructive uses the magnetic spin properties of atoms (most commonly, hydrogen) in a magnetic field to generate information on the water distributions and transitions, as well as the chemical and structural properties of food matrices to predict the dynamic changes that occur in food materials during processing (Thybo, Bechmann, Martens, & Engelsen, 2000; Thygesen, Thybo, & Engelsen, 2001).

Low field NMR has been widely used to evaluate the properties and potential uses of S*olanum tuberosum* (Irish potato) roots (Hansen et al., 2010; Mortensen et al., 2005; Thybo & Martens, 1999; Thygesen et al., 2001), positioning it as a potentially viable tool for studying other root and tuber crops. This potential has been verified by the authors in successfully determining the cooking characteristics of cocoyam cormels, flours, and starches in a study on the farmer varieties in Ghana (Boakye et al., 2017; Guðjónsdóttir, Boakye, Wireko‐Manu, Chronakis, & Oduro, 2016).

## 9 CHALLENGES TO ENHANCED COCOYAM UTILIZATION

The lack of policy and research interventions for the promotion and growth of cocoyam has relegated its production to the background compared with other root and tuber crops. To date, cocoyam production is still at the subsistence level in major growing areas (Acheampong et al., 2015; Onyeka, 2014), and farmers rely on traditional farming tools for production. As expected, the production levels are far below their estimated potential (Onyeka, 2014). This coupled with the required intensive weeding practice (usually requiring the assistance of expensive labor), has led to farmers using broad‐spectrum weedicides that steadily destroy the underground setts of cocoyam fields (Acheampong et al., 2015). The crop is no longer a volunteer crop (does not sprout after *burning and slashing* fields), but farmers need to acquire planting materials that are almost impossible to come by for lack of an organized market (Mr Kwame Affum; cocoyam farmer in Ghana, Personal Communication, 2014).

Access to improved varieties and microcredit is also challenges peasant farmers encounter every planting season (Acheampong et al., 2015). These drawbacks in production ultimately influence the prospects for industrial utilization. Compounding the drawback to enhanced utilization is the surrounding myths on its consumption, including the assertion that it can cause piles (as is assumed in Ghana), or that it is an ancestral food that should not be eaten by only one household but an entire community at all times (by the Krou people in particular, ‘Bete and Dida’ tribes in Côte d'Ivoire) (Anne Clarisse Sahe, Personal Communication, 2017), and it is regarded as a woman's/poor man's food in Nigeria (Onyeka, 2014). Other confounding factors include the difficulty in preparation and use of the edible portions, lack of diversity in existing dishes as well as paucity of information on their processing properties and potential alternate uses of the root. These challenges require serious attention to enable the transformation of a major root and tuber crop with great potential to addressing food security challenges within the subregion and the continent.

## 10 FUTURE TRENDS

Cocoyam, *Xanthosoma sagittifolium*, is a viable food commodity with appreciable nutritional profile, higher productivity, and better storability compared with other indigenous roots and tubers as well as having potential as a sustainable food security measure in the West African subregion. The cormels, flours, and starches could be explored in the snack and complementary food industries utilizing their peculiar processing properties, ease of crop production, and storability, as well as their nutritional value. The leaves, especially, are a food resource that requires attention from food technologists for it to be made available and ready‐to‐use all year. The potential health benefits could also be applied in the pharmaceutical industry. It is noteworthy that enhancing the food use of existing varieties and breeding new varieties with tailored end use is key to the continued cultivation and availability of cocoyam.

From the discourse, there is a need for continuous research interventions to explore the intricate molecular properties of the edible portions of *Xanthosoma sagittifolium* in seeking a better understanding of their mechanical and gelatinization properties for enhanced use. Although the existing indigenous use of cocoyam is currently limited, there are adaptable technologies and equipment to facilitate their exploitation in the snack, complementary, and general‐purpose industries, either as novel food products or improved indigenous foods. This calls for the concerted efforts of all stakeholders, especially crop breeders, food technologists, agricultural economists, food industries, and entrepreneurs to make this a reality. This notwithstanding, it is paramount for a taxonomic review of edible aroids in general, to facilitate information dissemination and use. This could be the first step to promoting the industrial and nonindustrial application of cocoyam on the global front.

## CONFLICT OF INTEREST

The authors have declared no conflict of interest.
